# Dietary strategies for improving iron status: balancing safety and efficacy

**DOI:** 10.1093/nutrit/nuw055

**Published:** 2016-12-13

**Authors:** Andrew M. Prentice, Yery A. Mendoza, Dora Pereira, Carla Cerami, Rita Wegmuller, Anne Constable, Jörg Spieldenner

**Affiliations:** *A.M. Prentice*, *D. Pereira*, *C. Cerami*, and *R. Wegmuller* are with the Medical Research Council (MRC) Unit The Gambia, Fajara, Banjul, The Gambia. *A.M. Prentice* and *R. Wegmuller* are with the MRC International Nutrition Group, London School of Hygiene & Tropical Medicine, London, United Kingdom. *Y.A. Mendoza*, *A. Constable*, and *J. Spieldenner* are with the Nestlé Research Centre, Lausanne, Switzerland. *D. Pereira* is with the Department of Pathology, University of Cambridge, Cambridge, United Kingdom. *C. Cerami* is with the Division of Infectious Diseases, Institute for Global Health & Infectious Diseases, University of North Carolina School of Medicine, Chapel Hill, North Carolina, USA.

**Keywords:** food fortification, iron, safety, staple foods, supplementation.

## Abstract

In light of evidence that high-dose iron supplements lead to a range of adverse events in low-income settings, the safety and efficacy of lower doses of iron provided through biological or industrial fortification of foodstuffs is reviewed. First, strategies for point-of-manufacture chemical fortification are compared with biofortification achieved through plant breeding. Recent insights into the mechanisms of human iron absorption and regulation, the mechanisms by which iron can promote malaria and bacterial infections, and the role of iron in modifying the gut microbiota are summarized. There is strong evidence that supplemental iron given in nonphysiological amounts can increase the risk of bacterial and protozoal infections (especially malaria), but the use of lower quantities of iron provided within a food matrix, ie, fortified food, should be safer in most cases and represents a more logical strategy for a sustained reduction of the risk of deficiency by providing the best balance of risk and benefits. Further research into iron compounds that would minimize the availability of unabsorbed iron to the gut microbiota is warranted.

## INTRODUCTION

Iron deficiency is estimated to be the most prevalent micronutrient deficiency worldwide and contributes to multiple pathologies mediated both through iron-deficiency anemia^1–3^ and through direct effects on the formation and function of organs, especially the brain.^4–6^ The reduction of iron-deficiency anemia is one of the six priorities of the World Health Organization’s (WHO’s) *Comprehensive Implementation Plan on Maternal, Infant, and Young Child Nutrition*.^7^ These priorities have been adopted as Priority Nutrition Indicators for the United Nation’s post-2015 Sustainable Development Goals.^8^^,^^9^

The highest prevalence rates of iron deficiency and iron-deficiency anemia occur in low-income countries where the combination of poor diet and high levels of infection chronically limits the uptake of iron. Because such populations can rarely afford iron-rich diets, especially those that contain the more bioavailable heme iron, the WHO recommends that governments of countries with a high prevalence of anemia implement programs of universal iron supplementation for young children and pregnant women.^10^^,^^11^

There are two problems with this approach: low levels of implementation, and the possibility that supplements may induce harmful side effects. In 2006, a significant excess of serious adverse events in African children receiving iron during a very large intervention trial^12^ raised serious concerns about the safety of iron supplements. These concerns have been reinforced by subsequent studies^13–15^ and are made plausible by the known stimulatory effect of iron on potentially pathogenic bacteria and protozoa.^16^ An expert committee convened by the WHO^17^ posited that it may be the nonphysiological nature of highly absorbable iron supplements, which bypass nature’s evolved systems for safely chaperoning iron and result in an excess of nontransferrin-bound iron, that underlies the harmful effects. The committee concluded that, although not yet proven, it was likely that iron delivered through the natural matrix of foods would be a safer alternative.

There are several methods currently in use for augmenting the level of dietary iron. Supplements (administered in the form of capsules or drops) are often used for the treatment of severe iron deficiency in a targeted population. Point-of-use fortification employs micronutrient powders in the form of packed, single-dose sachets that can be added to prepared food to improve its nutrient value. Biofortification involves the targeted breeding of staple food crops in order to increase their intrinsic content of micronutrients, including iron. Food fortification is the addition of micronutrients at the point of manufacture to enhance the nutritional content of the food items, such as meal ingredients or condiments. Unlike supplementation, iron fortification at the point of manufacture enables the delivery of small doses of the micronutrient in a food vehicle alongside the possibility of several servings per day. It is slower to raise body iron levels compared with iron supplementation or iron therapy, but it might be safer. The purpose of this review is to critically assess the latest evidence on the effects of dietary iron and the safety of augmenting iron levels using current strategies across all populations, including those from environments or settings with high levels of infection.

## IRON FORTIFICATION OF FOOD

Numerous studies have evaluated the effect of iron fortification in terms of health^18–20^ and economic benefits.^21^ Although many of the existing studies are based on food fortification in general, some have focused on specific packaged foods, such as beverages,^22^ milk, cereals,^18^ and condiments.^23^^,^^24^

Fortification of food products with iron implies the addition of iron-containing substances to the product recipe, either as isolated compounds (eg, iron salts or chelates) or as iron-rich ingredients (eg, meat or its derivatives). The choice of these ingredients is influenced by the desired product characteristics, including taste and color, and may be constrained by cost.

Although iron has been added to diverse food items since the early 1940s,^25^ its addition to packaged food products still poses technical challenges. Because of its oxidation-reduction properties, iron can create chemical instability in the food matrix, inducing organoleptic changes that are often unacceptable to the consumer.^26^ To overcome this technical obstacle, the industry uses insoluble, poorly soluble, or strongly chelated^27^ iron compounds, all of which have limited chemical reactivity. However, both solubility and chemical availability are necessary for effective absorption (bioavailability) of inorganic or nonheme iron. The preferred iron carriers must therefore offer a balance of these properties. These carriers must be tailored to the specific food product. For example, more soluble carriers can be used in dark-colored items (such as soy and fish sauces), which are less affected by color changes but are still prone to alterations in taste. Iron may react with other components of food products (such as unsaturated fatty acids, polyphenols, vitamins, etc), thereby affecting the shelf life and/or the nutritive value.

Beyond these technical issues, a key consideration is the bioavailability of the added iron. While heme iron is well absorbed in the human digestive tract, nonheme iron is generally poorly absorbed.^28^ Besides the differences in the mechanism of absorption of these two types of iron by the enterocytes – discussed elsewhere in this article – the absorption of nonheme iron is subject to strong interference from other common food components such as phytates from cereals^29^ and other staple foods (sorghum, pulses) or polyphenolic structures from fruits and vegetables. In fact, polyphenols can even inhibit the absorption of heme iron.^30^

The main iron compounds used for fortification of packaged foods are listed in Table 1, together with a qualitative assessment of their solubility and bioavailability (based on several references^26^^,^^27^^,^^31–34^).

**Table 1:** Main iron compounds used for the fortification of packaged foods

Compound	Iron load (% weight)	Solubility in water^a^	Relative bioavailability (%)^b^	Induction of taste, texture, or color changes	Approximate cost scale^c^
Ferrous sulfate 7H_2_0	20	High	100	High	Low
Dried ferrous sulfate	33	High	100	High	Low
Ferrous gluconate	12	High	89	High	High
Ferrous lactate	19	High	106	High	High
Ferric ammonium citrate	18	High	>100	High	Medium
Ferrous ammonium sulfate	14	High	≈100	High	Medium
Ferrous bisglycinate	20	High	>100	Low	High
Ferrous or ferric EDTA chelates	13	High	≥100 ^d^	Low	High
Ferrous fumarate	33	Low	100	Low	Low
Ferrous succinate	35	Low	92	Low	High
Ferric saccharate	10	Low	74	Low	High
Ferric glycerophosphate	15	Low	≈90	Low	Very high
Ferrous citrate	24	Low	74	Low	Medium
Ferrous tartrate	22	Low	62	Low	Medium
Ferric pyrophosphate	25	Low	21–74	Low	Low to medium
Ferric orthophosphate	28	Low	25–32	Low	Medium
Electrolytic iron powder	97	Negligible	75	Variable^e^	Very low
H-reduced iron	97	Negligible	13–148	Variable^e^	Very low
CO-reduced iron	97	Negligible	10–30	Variable^e^	Very low
Atomized iron	97	Negligible	ND	Variable^e^	Very low
Carbonyl iron	99	Negligible	5–20	Variable^e^	Very low

*Abbreviation*: CO, carbon monoxide; H, hydrogen; ND, not determined.

^a^Solubility can be enhanced in acid media.

^b^Compared with that of ferrous sulfate.

^c^Prices vary, depending on supplier.

^d^EDTA enhances the absorption of iron from other dietary components, especially in the presence of phytates or similar antinutrients.

^e^Dry products like cereals are less sensitive. Products with high water activity or acidity, like yogurt, are highly sensitive.

Since heme iron is readily bioavailable, there have been some instances of the use of meat-derived products in packaged food as fortificants.^35^^,^^36^ Although this could be a good approach for some populations that usually cannot afford sufficient quantities of meat in their diets, it may not be possible to extend it to more common food items because of issues related to consumer acceptance and/or price and availability.

Another natural source of iron is ferritin, a protein that can encapsulate high amounts of iron and is used by plants and animals for iron storage. It has been noted repeatedly that phytoferritin has high potential as a food fortificant.^37^^,^^38^ Currently, however, phytoferritin is not available commercially; therefore, it can be used only in products that contain sufficient amounts of foods that are naturally rich in this protein, such as pulses (beans, lentils, etc). Compounds that mimic the behavior of ferritin have recently shown very good bioavailability and are being explored for use in supplements,^39^ but they might be difficult to incorporate into some foods.

Manufacturers of fortified foods usually inform consumers of their products’ nutritional quality through label claims. In order to guarantee significant benefits for the consumer, regulatory bodies and health authorities define the minimum amounts of micronutrients that must be achieved before nutritional and/or health claims can be made. The WHO and Food and Agriculture Organization of the United Nations (FAO) *Codex Alimentarius*^40^ specifies that, in order to carry the nutritional claim of “fortified with iron,” products must contain at least 15% of the relevant Nutrient Reference Value (NRV) per measure of product. The claim “source of iron” requires that a product contain a minimum of 15% of the iron NRV (14 mg/d), ie, 2.1 mg per measure. The claim “rich in iron” requires that a product contain 30% of the NRV, ie, 4.2 mg per measure.

The *Codex Alimentarius*^41^ gives several options for determining the measure (Table 2). In general, these measures should be roughly equivalent. However, in the case of products that are consumed in small quantities but are nevertheless good carriers of fortification, such as condiments and bouillon cubes, the measures “100 g,” or “100 mL” or “100 kcal” are not realistic, since such quantities are not consumed in practice. Some companies estimate that the measure “per serving” is more appropriate to convey good nutrition,^42^ provided the serving is determined realistically and corresponds with the amounts typically consumed.

**Table 2:** Excerpt from the Codex Alimentarius *Guidelines for Use of Nutrition and Health Claims* (CAC/GL 23-1997)^41^

Component	Claim	Condition (not less than)
Vitamins and minerals	Source	15% of NRV per 100 g (solids)
		7.5% of NRV per 100 mL (liquids)
		or 5% of NRV per 100 kcal (12% of NRV per 1 MJ)
		or 15% of NRV per serving
	High	2 times the values for “source”

*Abbreviation*: NRV, nutrient reference value.

## BIOFORTIFICATION

Biofortification aims to provide staple food crops that are rich in micronutrients. By combining traditional breeding with modern techniques, biofortification blends the traits of high-yield crop varieties with high-iron varieties.^43^ In principle, the use of biofortified crops could address micronutrient deficiencies by enriching the staple food items that constitute the main portion of the diet in poor countries. Therefore, even very small amounts of micronutrients could have a positive impact over time. A second part of the reasoning is that, if biofortified crops also possess excellent agronomic characteristics, a self-sustaining public health intervention will result because farmers will favor such crops.

Iron biofortification is applicable to cereals like wheat, rice, and millet and to pulses like beans, peas, and lentils. The concentration of iron in regular crop varieties is relatively small: around 50 parts per million (ppm) in pulses,^44^^,^^45^ 25 ppm in wheat,^46^ and less than 15 ppm in rice.^47^ However, some wild or less common varieties contain amounts that are double or triple the average amounts. In grains such as wheat, iron is concentrated mainly in the outer layer (aleurone), which is usually separated in the bran and discarded in the process of making flour for bread, noodles, etc. Iron in the aleurone is most likely trapped in very tightly bound phytic acid structures.^48^ Logically, the high affinity of phytic acid to bind iron should hinder the use of iron by bacteria inhabiting the intestine. However, it is possible that phytases derived from various sources (plants or other dietary components), synthesized by the gut bacteria themselves, or produced by the intestinal mucosal cells can degrade phytates in the gastrointestinal tract and thereby free the chelated iron to some extent.^49^

The combination of low concentrations of iron in grains, interference from phytic acid, and separation of the bran during processing means that large amounts of wheat flour–based foods would have to be ingested in order for individuals to absorb sufficient quantities of iron.^50^ Iron-biofortified millet, which is grown in India and some other countries, contains higher concentrations of iron. Iron levels in this type of millet reach 90 ppm, whereas levels in nonbiofortified millet are around 20 ppm. Several studies indicate that regular intake of biofortified millet can be efficacious against iron deficiency.^51^^,^^52^

Biofortified pulses, containing 100 ppm or more, have the highest concentrations of iron.^53^ Although phytic acid is present in beans, a high proportion of the iron is contained in phytoferritin. Iron from ferritins has been shown to be highly bioavailable.^54^ Several studies have examined the bioavailability or efficacy of iron in biofortified beans consumed in developing countries.^55^ Generally, these studies have focused on the increase in iron absorption and the efficacy of biofortification, without giving consideration to possible negative outcomes or the undesired effects of iron on the microbiome (see sections below).

The current target levels for iron biofortification are in fact quite modest,^56^ far lower than those for iron supplementation. These targets have been established with staple crops (ie, foods consumed in large amounts, very frequently) and populations (rather than individuals) in mind. Higher levels may be reachable through genetic engineering, but genetically modified organisms remain stigmatized in many countries.^57^^,^^58^

## MECHANISMS OF IRON ABSORPTION: THE ROLE OF HEPCIDIN

Iron in foods can be broadly divided into heme and nonheme iron, of which the former is much more readily absorbed. The precise mechanism(s) by which heme iron is absorbed remains a matter of debate.^59^ In contrast, research over the past 2 decades has clarified the key elements of the molecular mechanisms by which nonheme iron is absorbed and by which all iron is distributed to tissues. Of central importance has been the discovery of hepcidin, the master regulator of iron homeostasis, and the elucidation of its mode of action in maintaining healthy body iron status.^60^ These basic science discoveries are of profound importance to the rational design of next-generation strategies for combating global iron deficiency and can inform predictions about the likely safety and efficacy of iron fortification programs.

Many detailed summaries of iron absorption and regulation are available elsewhere.^61^ In brief, soluble nonheme iron from the diet is reduced from Fe^3+^ to Fe^2+^ by a brush border ferric reductase, duodenal cytochrome B. The enterocyte then internalizes Fe^2+^ via the divalent metal transporter 1 (DMT1). It has been proposed that forms of nanoparticulate nonheme iron, such as the ferritin iron core, can be taken up whole by the enterocyte through endocytosis and are then dissolved only in vesicles inside the cell.^39^^,^^62–64^

Intracellular iron, derived from both pathways above, can then be stored (only temporarily, however, as the cells have a rapid rate of turnover) or transported across the basolateral membrane into the circulation. This process is facilitated by a single transmembrane transporter, ferroportin. The Fe^2+^ is then oxidized by the ferroxidases hephaestin and ceruloplasmin before it is taken up by the chaperone transporter transferrin for distribution to other organs and tissues.

Hepcidin regulates iron absorption by binding to ferroportin. When hepcidin binds to the extracellular arm of ferroportin, ferroportin is internalized and degraded, thus preventing iron egress. The iron blocked within the enterocytes initiates an intracellular regulation mechanism that further reduces the entrance of iron into the cell through divalent metal transporter 1; this blocked iron is ultimately excreted in the stool when the enterocytes die.

This new knowledge forces a radical reinterpretation of nutritionists’ concepts of iron absorption in several key respects. First, it has long been known that humans have no physiological means of regulating iron status through active excretion of iron, except for the loss of enterocytic iron that was never been fully absorbed. Iron is lost through menstruation or intentional or unintentional blood loss (trauma, childbirth, or therapeutic phlebotomy for iron overload), but these processes are unregulated. Therefore, the regulation of iron absorption is critical to the maintenance of healthy iron homeostasis. Furthermore, unlike regulatory processes in other metals such as zinc, whose efflux is regulated by multiple mechanisms, iron efflux from cells is regulated by a single mechanism, ie, ferroportin, which consequently allows precise control. Likewise, iron is the only trace element for which a hormone regulator, ie, hepcidin, has been discovered. As evidenced by these characteristics, iron absorption is a highly regulated process.

The earlier view held by nutritionists (and, hence, by policymakers) was that, when low iron levels in the body were due to low dietary intake of iron, the solution was to recommend large bolus doses of highly absorbable iron to be given between meals (to avoid the iron being bound to food phytates and related chelators) and, preferably, with vitamin C to enhance the reduction of iron from Fe^3+^ to Fe^2+^. This strategy works against the complex evolved mechanisms for the safe absorption and distribution of iron and may have caused significant iatrogenic disease (see below).

Further inferences from recent basic science discoveries relevant to iron fortification and supplementation derive from a new understanding of hepcidin regulation and from additional insights into genetic defects in iron pathways. Hepcidin is a downregulator of iron absorption and traffic, not an enhancer. It functions as the main inhibitor of iron transport from cells (including enterocytes, hepatocytes, and macrophages) into the blood circulation. At subthreshold levels of hepcidin, the divalent metal transporter 1 and ferroportin gates at both ends of the enterocytes are open, and iron is absorbed efficiently. Stable isotope studies in Gambian children have shown that iron absorption can be as high as 50% when hepcidin is switched off^65^ and is virtually zero at high levels of circulating hepcidin. Hepcidin levels are suppressed by iron deficiency and are elevated by either iron sufficiency/overload or inflammation.^66^ Two stable isotope absorption studies in Beninese women and Ivorian children demonstrated that iron absorption was doubled after asymptomatic malaria infection was cleared and hepcidin concentrations were reduced by approximately 50%.^67^^,^^68^

Therefore, hepcidin provides an elaborate system for the body to autoregulate iron influx by balancing the competing signals indicating a need for iron (hypoxia, erythroid drive, and hepatic iron deficiency) against the possible threat of an infection that might be fuelled by additional iron. In iron-deficient populations, hepcidin levels would tend to be low (to allow absorption) but are often high because systemic inflammation heralds the threat of infection. The interplay of these competing factors has been described in some detail for Gambian and Kenyan children.^69^ The take-home message for policymakers is that, rather than being unable to absorb adequate levels of iron, people living in unsanitary settings devote considerable physiological effort to *exclude* iron from their circulation. This is further validated by the tendency for all but one of the known genetic defects that alter iron status to lead to iron overload rather than deficiency, even in populations living on a low-iron diet.

## IRON, MALARIA, AND OTHER INFECTIONS

A decade ago, the WHO recommended that preschool children and pregnant women receive universal iron supplementation in countries in which the prevalence of anemia exceeded 40%. This included the great majority of low- and lower-middle–income countries. In practice, this advice was rarely implemented, and it was deemed necessary to gather stronger evidence of benefit to strengthen the arguments of advocacy. To this end, two very large intervention trials were commissioned in Pemba Island, Tanzania, and Nepal to compare the impact on overall mortality of iron plus folic acid; iron plus zinc; iron plus folic acid and zinc; and zinc alone against placebo in infants and young children. The trial in Tanzania was stopped early by the trial’s data safety monitoring board because there was evidence of a higher incidence of severe adverse events (hospitalizations from malaria and other infections and deaths) in the two arms receiving iron plus folic acid than in the zinc alone and placebo arms.^12^ The Nepali trial (conducted in a nonmalarious region) showed no such effect,^70^ and it was hence concluded that the adverse events in the trial in Tanzania had been caused by the interaction between iron and malaria. Following a series of expert consultations, the WHO and the United Nations Children’s Fund (UNICEF) issued a joint statement^17^ urging caution with regard to iron supplementation and recommending that supplementation be aimed at those who are anemic or at risk of iron deficiency. They further advised that supplementation be accompanied by strategies to prevent and treat malaria. Notably, food fortification was deemed to be safe and was explicitly excluded from this advice because “the patterns of iron absorption and metabolism may be substantially different.”^17^

The adverse outcome of the now infamous Pemba trial had the benefit of directing funding toward further field trials and generated a considerable body of basic research designed to elucidate the reasons behind the increased numbers of infections and deaths. It was widely speculated that nontransferrin-bound iron might be responsible. Under normal circumstances, all iron in the circulation would be tightly bound within transferrin and, hence, safely chaperoned to reduce the likelihood that it could be made available as nontransferrin-bound iron to any infectious organisms in the bloodstream. However, large nonphysiological bolus doses of iron can overwhelm the capacity of transferrin to collect iron, resulting in the formation of nontransferrin-bound iron.^71^ The potential link between nontransferrin-bound iron and infections is almost entirely based on conjecture. There are strong associations between percent transferrin saturation and the likelihood of systemic bacterial infections.^72^ Both gram-negative and gram-positive bacteria use two basic strategies to acquire iron from their human hosts: (1) chelation of iron away from chaperone proteins with siderophores; and/or (2) direct uptake of hemoglobin and heme moieties. Some bacterial species even lyse red blood cells to release the iron they need.^73^ There is also recent evidence that human blood collected 3 hours after consumption of an iron tablet supports greatly enhanced rates of replication of a number of pathogenic bacteria,^74^ but there is no evidence that nontransferrin-bound iron promotes malaria.

Subsequent to the Pemba trial, it has become clear that iron deficiency and iron-deficiency anemia are protective against malaria caused by *Plasmodium falciparum*. Most impressively, Gwamaka et al.^75^ monitored 785 children over a period of 3 years in Tanzania and showed that iron-deficiency anemia significantly decreased the odds of both subsequent parasitemia (23% decrease, *P* < 0.001) and severe malaria (38% decrease, *P*  =  0.04). This might operate at both the hepatic and the blood stages of the plasmodial life cycle. Studies in mice have shown that, during an initial infection, the *Plasmodium* parasite can protect itself from competition from another strain by utilizing the host’s hepcidin pathway to reduce iron in hepatocytes to such a level that the subsequent infection cannot establish a foothold.^76^ This prevents so-called superinfection and might reduce mortality in children. The mechanism is more potent when the host is iron deficient. Of probably greater relevance is the finding that iron-deficient red blood cells have a markedly reduced capacity to support plasmodial replication and growth.^77^ However, this protection is reversed by the presence of reticulocytes and young red blood cells, which are the favored niche for the merozoite stage. These latest insights indicate that the successful treatment of iron-deficiency anemia will always be accompanied by an increase in susceptibility to malaria, especially during the period of transient reticulocytosis required to augment the circulating red cell mass.^77^ This leads to two policy implications: first, that all strategies to combat iron-deficiency anemia (including food fortification) will inevitably cause a slight increase in the risk of malaria, and second, that aggressive supplementation strategies that result in rapid reticulocytosis will result in a transient period of greatly elevated risk (and, hence, should be accompanied by preventative malaria therapy).

Even before the Pemba trial, there was considerable evidence that administration of iron can enhance the risk of numerous other infections,^78–80^ but this had been overlooked by many investigators. More recent intervention trials have confirmed that the potentially harmful effects of iron are not limited to an increased risk of malaria and can also result from adding micronutrient powders containing encapsulated iron to foods at the point of use. A large cluster-randomized trial in a nonmalarious area of Pakistan reported a significant increase in the proportion of days with diarrhea (*P* = 0.001), bloody diarrhea (*P* = 0.003) and chest in-drawing (indicative of pneumonia) (*P* = 0.03) in children who received a micronutrient powder containing encapsulated iron (12.5 mg/d).^13^ A trial in Ghana revealed no apparent increase in the incidence of malaria but a significant increase in hospitalizations during the period when sprinkles containing 12.5 mg of iron were administered.^14^ The Chilenje Infant Growth, Nutrition, and Infection Study (CIGNIS), which provided richly micronutrient-fortified porridge (12.5 mg iron) to 6-month-old Zambian infants, revealed an increase in the incidence of lower respiratory tract infections at scheduled clinic visits (*P* = 0.02).^81^ Similarly, a study that administered micronutrient powders with a low iron content (2.5 mg as sodium iron EDTA [NaFeEDTA]) to Kenyan infants has reported that infants in the iron group spent significantly more days with cough and dyspnea.^82^ In a recently completed trial of physician-prescribed lipid-based multiple micronutrients in The Gambia,^83^ the supplements failed to reduce the frequency of repeat clinic visits and, on secondary analysis, revealed an increase in repeat visits in the first 3 weeks of administration in those receiving the fortified lipid-based nutrient supplements.^84^ A study in Cambodian schoolchildren using 3 different types of micronutrient-fortified rice (all containing ≈8 mg of iron per portion) has demonstrated that micronutrient-fortified rice significantly increased the risk of new hookworm infection.^85^ In each of these trials, iron was given with multiple other micronutrients, so it is not certain that iron caused the pathology, but, on the basis of prior evidence about the importance of iron to microorganisms, this is the widely held assumption.

Data from these last trials raise potential concerns, even about point-of-use fortification with iron, although it should be noted that the amounts of iron used in these trials were higher than those achieved through point-of-production fortification of staple or other types of foods.

## EFFECTS OF IRON ON THE GUT MICROBIOTA

The latest research is beginning to shed light on the effects of dietary components, including iron, on the microbial communities of the gut. The microbiota of the human gut contains a diverse spectrum of bacteria, viruses, and bacteriophages all competing for survival and niche occupancy.^86^^,^^87^ Under optimal circumstances, the human host and its microbiome form a mutually supportive and unique symbiotic partnership^88–90^ that ensures good health for the host.^91^ As described above, most bacteria have an obligate need for iron and have evolved a myriad of biological strategies to obtain iron, which is made scarce not by its absence but by its very low solubility in the more common ferric state. The wide diversity and effectiveness of these bacterial iron acquisition mechanisms is a testament to the critical importance of iron for the survival and success of bacteria.

The bacterial species considered most beneficial to the host (members of the *Lactobacillaceae* and *Bifidobacteriaceae* families) are unusual in having either no requirement or a very low requirement for iron.^92^ The human host has developed elaborate strategies that can favor the growth of beneficial bacteria. Human milk has a very low iron content, and lactoferrin in milk tightly binds iron as a mechanism to limit iron. It is also probable that any iron taken up by enterocytes and encased in ferritin will be largely unavailable to potentially pathogenic bacteria in the distal large intestine and colon, even if it is not absorbed into the body and re-enters the gut in sloughed enterocytes. If this is the case, then the major source of iron for bacteria in the gut will be unabsorbed soluble forms of iron, which will be found in the highest concentrations when excess iron is consumed (as in the case of therapeutic supplementation).

These theoretical predictions are borne out by in vitro, animal, and human clinical studies,^93^ highlights of which are summarized here. First, evidence that iron administration is associated with an increase in the prevalence of diarrhea was summarized in the previous section. The effect of iron on the spectrum of bacterial species in the gut has also been investigated. Zimmermann et al.^94^ described a decrease in beneficial lactobacilli and bifidobacteria and an increase in potentially pathogenic enterobacteria in stool samples from Ivorian schoolchildren who received biscuits fortified with iron. The group receiving iron also had much higher levels of fecal calprotectin, a marker of gut inflammation.^95^ Very similar results were obtained in Kenyan infants who received porridge fortified at home with 2.5 mg of iron as Fe-EDTA or 12.5 mg of iron as ferrous fumarate when compared with placebo controls who received the same multiple micronutrient powders, but without iron.^96^ The effect on fecal calprotectin was particularly striking and is also concerning because environmental enteropathy (persistent gut damage and inflammation that leads to malabsorption) is a major cause of growth failure in children in poor environments. The CIGNIS trial in Zambia also found a significant increase in the ratio of lactulose to mannitol (an index of gut permeability) in human immunodeficiency virus (HIV)-exposed and -unexposed young children who received a micronutrient-fortified porridge compared with those who received the unfortified porridge.^97^

These human clinical studies have been supported by results from in vitro culture systems demonstrating that the addition of small amounts of iron to kinetic models of human gut metabolism causes a shift toward a more toxic profile within the microbiota.^98–99^ Although the long-term clinical relevance of these findings is still unknown, these data suggest that the influence of iron on the gut microbiota is an important factor that needs to be evaluated in studies investigating the effects of any dietary intervention.

## IRON AND BLOOD DISORDERS

### 

Any program for universal fortification of foods needs to consider whether there might be subsections of the recipient population who could be harmed by the addition of the nutrient. For instance, the United Kingdom has not adopted folate fortification, partly due to concerns that additional folate might promote tumor growth in cancer patients or conceal pernicious anemia in the elderly. The United States, on the other hand, has adopted folate fortification.

In the case of iron, an important consideration is whether there are circumstances in which recipients could become iron overloaded to an extent that would damage their health with life-threatening consequences (primarily through induction of cirrhosis, diabetes, or cardiomyopathy). There are a number of clinical conditions in which this might be a concern, notably hemochromatosis, beta thalassemia, and repeated blood transfusions.

#### Hemochromatosis.

Hemochromatosis is the generic term for iron overload, of which the two leading causes are hereditary hemochromatosis and transfusional iron overload (see below). Hereditary hemochromatosis, which has various genetic causes with differing clinical manifestations, leads to iron overload and subsequent toxicity, especially in the liver. Treatment is possible if the condition is recognized early. Hereditary hemochromatosis is most common in people of Celtic, English, or Scandinavian origin, in whom the prevalence is between 0.5% and 1%.

#### Beta thalassemia.

Beta thalassemia is a genetic defect that prevents the production of the beta chain of hemoglobin and leads to microcytic anemia, which causes a reticulocytosis as the body attempts to compensate. This in turn causes the downregulation of hepcidin (mediated by the newly discovered erythroferrone), which leads to iron overload. Patients require regular blood transfusions, which further contribute to the accumulation of iron. If not treated by iron chelation, the resultant iron overload can cause myocardial siderosis and death from heart failure. Beta thalassemia affects about 1 in 100 000 people globally.

#### Transfusional iron overload.

A number of conditions that require regular blood transfusion (including beta thalassemia) can lead to secondary iron overload. Though serious for the individuals affected, such conditions are rare, and the danger of iron overload can be ameliorated by chelation therapy.

## POTENTIAL ROLE OF IRON IN OTHER CIRCUMSTANCES

Numerous articles about the role of iron in neurodegenerative diseases have been published, several of which are included in this review.^100–102^ Although a link between iron accumulation and neurodegenerative disease has been recognized, the complete mechanisms have not been elucidated. There are many factors that could potentially trigger these complex diseases, including the presence of other transition metals such as copper, zinc, and aluminium.^103^^,^^104^ For the purpose of this review, there is no evidence that intake of iron within the recommended dietary allowances could be a direct cause of neuropathies.

A follow-up study of 10-year-old Chilean children who participated as infants in a randomized controlled trial of high-iron vs low-iron formulas is sometimes cited as demonstrating significantly adverse outcomes at 10 years of age for a few of the psychometric measures,^105^ but this conclusion is based on a post-hoc subanalysis that resulted in a very small sample size and, hence, requires replication.

Similarly, iron has been associated with adverse cardiovascular effects,^106^ although the simple positive association observed in cross-sectional epidemiological studies does not confirm causality. In cases of transplantation of heart or kidney, both iron deficiency and overload may lead to complications.^107^ However, as far as can be determined, there is no information that would link food fortification to any of these problems.

The relationship between iron and some cancers has been considered frequently in the last half century. Research for cancer treatments shows that iron could act both as a factor of carcinogenesis^108^ and as a cytotoxic agent for cancerous cells.^109^^,^^110^ The risk of colorectal cancer could be connected to heme iron consumption,^111^ but there is no conclusive link with total dietary iron.^112^ After considering data from numerous studies, the International Agency for Research on Cancer recently examined the contribution of heme iron from meat intake to increased risk of cancer.^113^

For type 2 diabetes, epidemiological observations and experimental studies show a clear link between iron overload and risk of diabetes.^114^ Here again, from a nutritional perspective, only high heme iron intake showed a significant association with type 2 diabetes. Total dietary iron, nonheme iron, and even supplementation are not significantly associated with the risk of diabetes.^115^

Complex interactions between iron deficiency, malaria, and HIV infection have been reported.^116^^,^^117^ Anemia is highly prevalent in pediatric populations in Africa and Southeast Asia, and in many countries there is overlap between patients infected with HIV and malaria, and anemia. Treating iron deficiency can result in improved survival of HIV-infected children,^117^ but – as noted previously in this review – the additional intake of iron can also favor malaria. Consequently, it is difficult to decide whether a population of children at risk of both HIV and malaria infections would benefit from iron supplementation unless there is simultaneous provision of malaria treatments or precautions (such as insecticide-treated bed nets). Joint infection with HIV and *Mycobacterium tuberculosis* can promote anemia and iron redistribution, but again, additional iron intake may not be safe.^118^ During the preparation of this review, no published data was found about iron fortification (low doses) in patients with HIV infection, HIV infection and malaria, or bacterial infections.

## BALANCING THE RISK AND BENEFITS OF ADDITIONAL IRON INTAKE

The intensive global efforts to combat iron deficiency and its associated anemia are predicated on the belief that iron plays a crucial role in human health, especially in early child development, since there is evidence of critical windows of time beyond which any deficits in brain development cannot be rectified.^4–6^ On the basis of this concern, the remainder of this review will focus more on the potential negative effects of iron.

The safety concerns related to the provision of excess iron to populations with low baseline health status (such as those in malaria-endemic areas) call for the adoption of alternative strategies. A central question is whether to use a targeted approach, ie, by providing iron only to those who are clearly iron deficient, or to provide iron to all those in whom iron deficiency presents the greatest health risks (eg, infants, young children, and pregnant women). A major barrier to adopting the targeted approach is the detection of iron deficiency. Aside from the lack of established biomarkers of iron status that can be easily applied in the field, screening for iron status at the population level is challenging. Another issue is that supplements and point-of-use fortification are short-term measures better suited as targeted interventions in a defined group rather than as long-term strategies that can be implemented at the population level.

These considerations, when taken together with current knowledge of the potential adverse effects of iron in low-income populations at risk of disease, point toward the use of very low doses of iron that can be delivered consistently. To this end, food fortification presents a potential strategy to mitigate the effects of iron deficiency across a population. In developed countries, nationwide food-fortification programs have been well established. Folic acid fortification, for example, has played a key role in reducing birth defects in Canada, the United States, and Chile and is a good strategy for reaching the population of women at risk of deficiency.^119^^,^^120^ The ingestion of small amounts of iron with food provides an opportunity to minimize circulating levels of nontransferrin-bound iron or high concentrations of unabsorbed iron in the gut.

A randomized, placebo-controlled, double-blind intervention trial performed a head-to-head comparison between iron supplements and food fortification in 425 Vietnamese school-aged children.^121^ Results showed that, although both interventions yielded significant improvements over placebo in terms of hemoglobin, ferritin, and body iron levels and anemia status, the increases in the fortification group were less pronounced compared with those in the supplement group. These differences were most likely due to the lower amounts of iron received with fortification. After comparing the effectiveness of both approaches, the authors concluded that, in a population of anemic children with mild iron deficiency, iron fortification should be the preferred strategy over the longer term.

Infants and young children are among those at greatest risk of not only iron deficiency but also the developmental consequences of iron deficiency. Although infants aged 6 to 24 months are not often included in global estimates of iron-deficiency anemia, current evidence indicates that this group is at particularly high risk.^122^ Beyond 6 months of age, an infant’s need for iron begins to exceed the amount provided by breast milk.^123^^,^^124^ This is more pronounced in infants who show rapid growth, such as low-birth-weight infants who undergo catch-up growth.^122^ A recent systematic review of 18 trials found that the use of milk and cereal products fortified with iron plus multiple micronutrients increased hemoglobin levels by 8.7 g/L in children aged 6 months to 5 years and reduced the risk of anemia by 57% compared with the use of nonfortified items.^18^ The introduction of iron-rich complementary foods has also shown a beneficial effect on the iron status of infants.^125^ The Chilean National Complementary Feeding Program showed that iron-fortified milk improved iron status and reduced the prevalence of anemia over the long term in children aged 11 to 18 months. The prevalence of anemia was 27% prior to the introduction of the program but was reduced to 9% 1 year after the introduction of the program (*P* < 0.001).^126^ After adjusting for confounding factors, the consumption of iron-fortified milk was associated with higher hemoglobin concentrations and a lower prevalence of anemia. The benefits were apparent 10 years after the implementation of the program, suggesting long-term, sustained effects on iron status and the prevalence of anemia in children.

Thus far, there has been no data demonstrating specific adverse effects of iron-fortified food items. In their systematic review, Eichler et al.^18^ suggest that the recommendations for iron supplements may not apply to fortified foods, since the daily dose of micronutrients obtained with fortified foods is far lower than that obtained with supplementation. There are still uncertainties about the hemoglobin cutoff values used to define anemia in children and in specific populations such as those with hemochromatosis, beta thalassemia, and repeated blood transfusions. Thus far, most studies have used iron status, hemoglobin levels, or anemia as measures of efficacy of iron fortification. Long-term functional outcomes – such as growth, cognitive development, morbidity, economic productivity – are more difficult to measure, but additional data on these will be the true measure of the long-term safety and efficacy of any intervention. Long-term data on the use of fortified foods in populations with a high disease burden (including malaria and other infectious diseases) are thus needed to determine the best means of addressing iron deficiency at the population level.

## CONCLUSION

The evidence summarized here leads to several conclusions. First, new insights into human iron metabolism, especially those derived from the discovery of the hepcidin/ferroportin system for regulating iron uptake, suggest that the strategy of providing large nonphysiological bolus doses of highly absorbable iron as supplements works against the highly evolved systems for safely maintaining iron homeostasis in humans and, hence, may be hazardous. Second, an important cause of iron deficiency is the inflammatory blockade, and any strategy to reduce iron deficiency and iron-deficiency anemia should include interventions to limit infections and inflammation (including low-grade inflammation). If inflammation can be reduced, then elimination of the iron blockade will permit healthy iron homeostasis at lower levels of dietary iron. Third, chemical or biological fortification of staple foods provides a logical approach as a public health strategy and should be pursued. The data do not support the conclusion that iron-fortified foods are without risk, but any possible risks are probably much lower than those associated with the high burden of disease and reduced functional capacity caused by iron deficiency worldwide. When cost or any other constraint prevents treatment of inflammation or infection, fortification with low doses of iron homogeneously diluted in a larger mass of food remains one of the safest strategies available to reduce the risk of deficiency. These considerations are important in the context of the United Nations Sustainable Development Goals, since iron-deficiency anemia is a main indicator of micronutrient deficiencies among women of reproductive age and children under the age of 5 years. Future research is required to examine the effects of unabsorbed iron on the gut microbiota. Further testing of compounds containing iron that is absorbable in the human duodenum but is unavailable to potentially pathogenic bacteria in the large intestine and colon is warranted.
